# Hypnotic induction is followed by state-like changes in the organization of EEG functional connectivity in the theta and beta frequency bands in high-hypnotically susceptible individuals

**DOI:** 10.3389/fnhum.2014.00528

**Published:** 2014-07-24

**Authors:** Graham A. Jamieson, Adrian P. Burgess

**Affiliations:** ^1^School of Behavioural, Cognitive & Social Sciences, University of New EnglandArmidale, NSW, Australia; ^2^Aston Brain Centre, School of Life & Health Sciences, Aston UniversityBirmingham, UK

**Keywords:** hypnosis, EEG, theta rhythm, beta rhythm, functional connectivity, coherence, imaginary coherence

## Abstract

Altered state theories of hypnosis posit that a qualitatively distinct state of mental processing, which emerges in those with high hypnotic susceptibility following a hypnotic induction, enables the generation of anomalous experiences in response to specific hypnotic suggestions. If so then such a state should be observable as a discrete pattern of changes to functional connectivity (shared information) between brain regions following a hypnotic induction in high but not low hypnotically susceptible participants. Twenty-eight channel EEG was recorded from 12 high susceptible (highs) and 11 low susceptible (lows) participants with their eyes closed prior to and following a standard hypnotic induction. The EEG was used to provide a measure of functional connectivity using both coherence (COH) and the imaginary component of coherence (iCOH), which is insensitive to the effects of volume conduction. COH and iCOH were calculated between all electrode pairs for the frequency bands: delta (0.1–3.9 Hz), theta (4–7.9 Hz) alpha (8–12.9 Hz), beta1 (13–19.9 Hz), beta2 (20–29.9 Hz) and gamma (30–45 Hz). The results showed that there was an increase in theta iCOH from the pre-hypnosis to hypnosis condition in highs but not lows with a large proportion of significant links being focused on a central-parietal hub. There was also a decrease in beta1 iCOH from the pre-hypnosis to hypnosis condition with a focus on a fronto-central and an occipital hub that was greater in high compared to low susceptibles. There were no significant differences for COH or for spectral band amplitude in any frequency band. The results are interpreted as indicating that the hypnotic induction elicited a qualitative change in the organization of specific control systems within the brain for high as compared to low susceptible participants. This change in the functional organization of neural networks is a plausible indicator of the much theorized “hypnotic-state.”

## Introduction

Hypnosis here refers to a group of practices in which suggestions are employed to bring about desired changes in behavior, experience and physiology similar to what might be expected if the suggested events were real. These suggestions are preceded by a clearly designated hypnotic induction ritual, which marks them out from mundane reality, and terminated by a hypnotic de-induction, which marks the return of everyday experience. Hypnosis is widely used to control pain and distress in a variety of clinical settings and provides empirically supported treatments for a number of important medical conditions and empirically promising treatments for many more (Mendoza and Capafons, 2009). Hypnotic susceptibility, the ability to respond to hypnotic suggestion, is reliably measured by administration of standardized scales, comprised of specific suggestions tapping a wide range of traditional content areas: ideomotor (involuntary movement) suggestions, various forms of motor paralysis, positive sensory hallucinations, negative hallucinations (blockage of particular experiences such as in hypnotic analgesia), transformations in aspects of the self (e.g., age regression), or post hypnotic amnesia (Woody and Barnier, 2008). Specific test suggestions employ objective response criteria and have well known difficulty levels.

From the inception of scientific investigations into hypnosis (the report of the Royal commission led by Benjamin Franklin in 1784) down to the present day, one central question has divided scientific researchers in the field. That is, are the profound hypnosis-induced changes in experience reported by highly susceptible individuals the result of a similarly profound shift in the operation of the mind-brain system or can they be explained by the operation of mundane psychological processes such as imagination, attention and response expectancies (Kihlstrom, 2002; Lynn and Lilienfeld, 2002)? Recently a variety of specific hypnotic suggestions have been employed in neuroimaging studies making important contributions to the cognitive neuroscience of volition, motor control, attention and pain perception; researchers are now poised to extend these investigations to address the delusions found in a range of clinical neuropsychological conditions (see the comprehensive Nature Neuroscience review by Oakley and Halligan, 2013). While these studies address the role of specific psychological mechanisms in specific suggestions they do not address (or seek to address) the possibility of a fundamental shift in the operation of the mind-brain system in hypnosis. Given that the electroencephalogram (EEG) has been able to show that specific neurophysiological processes are associated with the phenomenologically distinct states of experience (for operationalization of this construct see, Tart, 1975; Pekala, 1991) found in specific states of arousal, attention, epilepsy, sleep stages (Dement and Kleitman, 1957), dreaming (Aserinsky and Kleitman, 1953) and coma (Boccagni et al., 2011) it is not surprising that many researchers have sought to use the EEG to address this issue (Sarbin and Slagle, 1979; Perlini and Spanos, 1991; Fingelkurts and Fingelkurts, 2014).

Many studies have reported spectral band-power changes between pre and post the hypnotic induction, or between high and low susceptibles, or in relation to specific hypnotic suggestions (particularly analgesia) most commonly in the theta (Blais et al., 1990; Sabourin et al., 1990; Crawford, 1994; Graffin et al., 1995), upper alpha (Williams and Gruzelier, 2001; Terhune et al., 2011) and gamma (De Pascalis, 2007) frequency bands. It is not our purpose to review this work here but differences in method, inconsistent findings and the absence of replication prevent any firm conclusion being drawn (Lynn et al., 2007).

However, whereas other phenomenologically distinctive states of consciousness (sleep stages, dreaming, coma etc.) can be recognized from their characteristic EEG profiles, there is no such distinguishing feature for hypnosis and the changes in the EEG reported during hypnosis are well within the range of what is seen during normal non-hypnotic conditions. So, even though these hypnosis-related changes in the EEG might provide important evidence about the nature of the neural mechanisms involved, they do not constitute the sort of qualitatively distinct difference that seems to be required to support the “altered state” interpretation of hypnosis (Hasegawa and Jamieson, 2002; Burgess, 2007). Furthermore, given the large number of studies that have measured EEG during hypnosis, it is reasonable to conclude that, if a hypnosis-specific pattern of EEG band-power had existed, it would have been found long ago.

This is not to suggest that the usefulness of the EEG in this context has been exhausted but that we may have been looking in the wrong place. Phenomenologically distinct states do not seem to be characterized by localized brain activity but by the pattern of interactions between multiple spatially separated neural assemblies (see e.g., Tononi and Edelman, 1998). Conscious experience then, arises from the activity of multiple local cortical sources that interact in a constant flux of mutual influence and informational exchange through cortico-cortical white fiber pathways, through cortico-thalamo-cortical pathways and finally through cortico-striatal-thalamo-cortical pathways in rapidly forming and dissolving networks of functional coalitions (Kelso, 2012; Fingelkurts et al., 2013). The shared information that constitutes these functional neural networks is primarily expressed in the phase or timing relationships between recorded oscillations. It is in this “deep structure” (a form of latent mathematical description) which represents the functional core of the EEG rather than the surface structure expressed in spectral band power/amplitude that the hypnotic state, if it exists, can be expected to be found (Burgess, 2007). It is this aspect of the EEG, functional connectivity, which we address in the current study in order to seek evidence for a possible marker for what may be termed “the hypnotic state.”

While there are numerous measures of functional connectivity available to cognitive neuroscience researchers the most widely studied and best understood such measure employed in EEG research is coherence (COH) which provides an index of the frequency specific phase consistency between two time series (typically derived from separate electrodes; Shaw, 1984). Coherence analysis has been employed sporadically in hypnosis research since the 1990's (Sabourin et al., 1990; Kaiser et al., 1997) but across widely divergent paradigms and without consistent findings. Significant decreases in gamma band COH have been reported in high susceptible hypnotized participants; between frontal and somatosensory electrodes in the case of hypnotic analgesia (Trippe et al., 2004). Hypnosis-related decreases in gamma coherence have also been reported and between frontal midline and left fronto-lateral electrodes during the Stroop task suggesting a breakdown in functional connectivity between functionally related locations of the frontal cortex and other regions (Egner et al., 2005).

However, all existing studies of EEG COH and hypnosis share two major problems of interpretation: volume conduction and inflated Type-1 error caused by multiple comparisons. Volume conduction means that electrical activity from a single source may be detected at multiple electrode sites (Fein et al., 1988) which can seriously inflate coherence between the channels. There are a number of functional connectivity measures able to address this issue (see e.g., Stam et al., 2007) but for this study we adopted the imaginary component of coherency (iCOH; Nolte et al., 2004—see Materials and Methods for details). The second problem, that of an inflated Type-1 error arises because for *n* EEG channels, there are *n*(*n* − 1)/2 possible channel pairings requiring multiple statistical comparisons and some appropriate method of Type-1 error control. In this study, we used a multivariate method of analysis, Partial Least Squares (PLS) (Lobaugh et al., 2001; McIntosh and Lobaugh, 2004), that not only controlled the overall Type-1 error rate but also allowed us to identify the “deep structure” of the differences in functional connectivity between the hypnotic and pre-hypnotic states.

As understood here a “hypnotic state” corresponds to a qualitative restructuring of the operational framework in which psychological processes take place and accounts for the distinctive phenomenology of the hypnotized person (Pekala and Kumar, 2007; Cardeña et al., 2013). This state is hypothesized to be initiated, in those susceptible to hypnosis, by the hypnotic induction and facilitates, if not enables, the operation of distinct psychological processes which implement responses to specific hypnotic suggestions such as amnesia, age regression or hypnotic analgesia (Mazzoni et al., 2013). If hypnosis brings about a change in mental functioning that enables or facilitates the operation of the processes which underlie the response to specific types of hypnotic suggestion then we would expect to see evidence of this as a change in the organization of functional connectivity in the hypnotic condition following a hypnotic induction but prior to the administration of specific hypnotic suggestions. However, as first articulated by the late Ken Bowers, no response, whether behavioral, experiential or physiological, elicited by a hypnotic procedure may legitimately be termed hypnotic unless it is associated in some way with the participants' measured level of hypnotic susceptibility (Woody, 1997).

The aim of the present study was to investigate EEG functional connectivity recorded during eyes closed resting before and after a standard hypnotic induction procedure in groups of high and low susceptible participants respectively. Functional connectivity was measured using COH and iCOH and PLS was employed to identify any components (deep structure) with a significant relationship to the interaction of state (pre-hypnosis vs. hypnotized) and trait (high vs. low susceptible) conditions of the experimental design.

## Materials and methods

### Participants

Participants were recruited from students at Imperial College London who were pre-screened using the Harvard Group Scale of Hypnotic Susceptibility: Form A (HGSHSA) (Shor and Orne, 1962). A subset of high (HGSHSA score ≥9) and low (HGSHSA score ≤3) scorers were invited to individual screening using the Stanford Hypnotic Susceptibility Scale Form C (SHSSC) (Weitzenhoffer and Hilgard, 1962) to confirm their hypnotic susceptibility. Those who continued to score ≥9 on the SHSSC were identified as high susceptibles and those who continued to score ≤3 on the SHSSC were identified as low susceptibles. The final sample consisted of 12 high susceptible participants (age range 20–24, 2 men) and 11 low susceptible participants (age range 20–24, 3 men). All participants were healthy and right handed. Written informed consent was obtained from all participants and the experiment was conducted as approved by the Riverside Research Ethics Committee and followed the principles expressed in the Declaration of Helsinki and data were analyzed anonymously.

### Procedure

**Figure 1:**
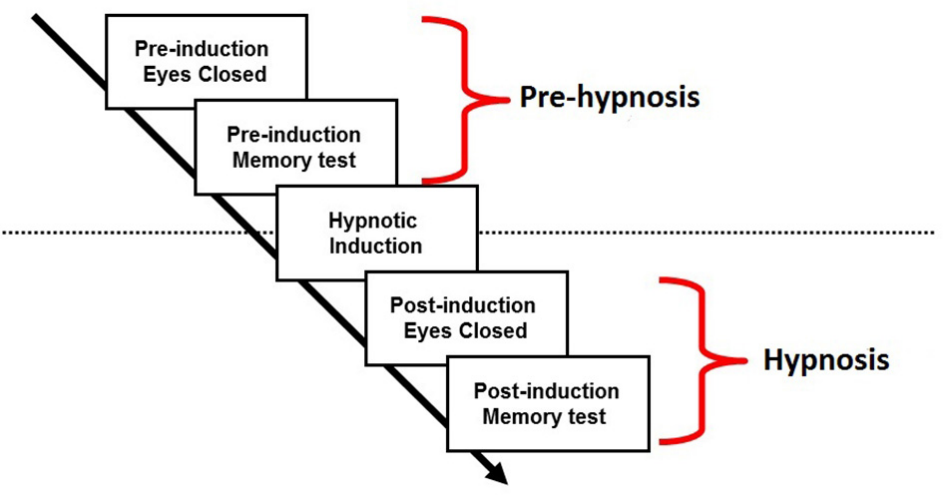
**Procedure**.

The procedure of the experiment is outlined in Figure 1. EEG was recorded from participants as they sat with their eyes closed (4 min) followed by a continuous recognition memory test for words and faces (15 min). This period will be referred to as the “pre-hypnosis” state. They then underwent the standard hypnotic induction procedure from the SHSSC (fixation on a visual target followed by eyelid heaviness and involuntary eye closure) and sat with their eyes closed for a further 4 min followed by a second memory test. This period will be referred to as the “hypnosis” state. The data to be reported in this paper refer to the EEG recorded during the resting state with the eyes closed in the pre-hypnosis and hypnosis conditions. Analysis of the recognition memory paradigm will not be reported here.

### Materials and equipment

EEG was recorded using a 32 channel Neuroscan Synamps amplifier. Signal bandpass was 0.1–100 Hz and the digital sampling frequency was 500 Hz. Twenty-eight electrodes were positioned on the scalp using an ECI electrode cap with electrodes placed according to the International 10–20 system with an additional nine electrodes: Oz, FC5/6, CP1/2, CP5/6, and PO1/2. Electrode impedances were all under 5 kΩ. Reference was to the left ear and converted to average reference offline.

### Signal preparation

EEG was divided into consecutive segments of 2048 ms and detrended. Any epochs including values outside of range −100 μ V to +100 μ V range were excluded from further analysis. The acceptance rate for epochs was high across all participants and in the worst case was 83%. All analysis was performed on these epochs which, with duration of 2048 ms and a sampling rate of 500 Hz, gave frequency resolution of 0.488 Hz.

#### EEG power spectrum

The power spectrum of the EEG at each channel was estimated in the 0.488–44.921 Hz frequency range using FFT following Welch's method with a Hamming window. Power values were converted to amplitude values by taking the square root of the power as amplitude follows an approximately normal distribution.

#### EEG functional connectivity

Functional connectivity between all 28 channels was measured using coherence (COH) (Shaw, 1984) and imaginary coherence (iCOH) (Nolte et al., 2004). COH is a widely used measure of functional connectivity in EEG research and is a normalized measure of the phase consistency between two signals that ranges from 0 to 1. However, COH provides an inflated estimate of the true functional connectivity because it is susceptible to the effects of volume conduction. Volume conduction means that electrical activity from a single source may be detected at two spatially separate recording sites and, unfortunately, COH is unable to distinguish between the case of a single common source and functional connectivity between two or more distinct sources.

Fortunately, there are several simple measures of functional connectivity that are insensitive to the effects of volume conduction and we chose one of those recommended by Stam et al. (2007), iCOH. COH is the absolute value of coherency (COHy), which is a complex number (made up of a real and an imaginary component: rCOHy and iCOHy) that represents the normalized cross-spectrum of the two signals of interest. The real component, rCOHy, represents that part of the co-variation between the signals that is zero-phase lagged (i.e., instantaneous) whereas the imaginary component, iCOHy, represents the part that is phase-lagged. As the effects of volume conduction are always instantaneous (i.e., zero-phased), iCOHy provides an index of functional connectivity that is insensitive to the effects of volume conduction. However, zero-lagged connectivity may not all be due to the effects of volume conduction, excluding rCOHy means that some real connectivity will be excluded also meaning that iCOHy will provide an underestimate of the true connectivity. For convenience, instead of using iCOHy, we used the absolute value which is imaginary coherence, iCOH. Like COH, iCOH is a normalized measure of connectivity that ranges from 0 to 1. In short, we used two estimates of functional connectivity: COH, which overestimates the “true” connectivity as it includes the effects of volume conduction and iCOH, which is insensitive to the effects of volume conduction but which underestimates the “true” connectivity because it will exclude any real zero-lagged effects.

COH and iCOH were estimated following Welch's method with a Hamming window and averaged across frequency bands: delta (0.1–3.9 Hz), theta (4–7.9 Hz) alpha (8–12.9 Hz), beta1 (13–19.9 Hz), beta2 (20–29.9 Hz), and gamma (30–45 Hz) frequency ranges. With 28 channels this gave a total of 378 electrode pairs.

### Statistical analysis

#### EEG power spectrum

FFT amplitude spectrum data (0.488 to Hz 44.921 in steps of 0.488 Hz) from the high and low susceptible groups for the pre-hypnosis and hypnosis conditions were compared using PLS analysis(Lobaugh et al., 2001). The PLS analysis was performed in MatLab using a software package available from http://www.rotman-baycrest.on.ca/. PLS is a method for determining whether the values of a multivariate dataset are systematically affected by the experimental manipulation, in this case, STATE (i.e., Hypnosis vs. Pre-hypnosis) and/or GROUP membership (High vs. Low susceptible). PLS extracts a series of latent variables (LV) that maximally differentiates the covariances in the data according to the experimental design and group membership. This is done by singular value decomposition of the crossblock covariance matrix (i.e., the cross-product of the design matrix and the data matrix). The relative importance of each LV is indicated by the percentage of the crossblock covariance matrix that it can account for and the statistical significance of each LV is determined by permutation testing. In this case, with two experimental conditions and two groups, a total of four LVs will be extracted but only the first two will be meaningful. The meaning of each LV can be determined by examination of the Design Scores which indicate the relative weighting of each on the four conditions (Pre-hypnosis & High-susceptible, Hypnosis & High-Susceptible, Pre-hypnosis & Low-susceptible and Hypnosis & Low-Susceptible). PLS also produces “saliences” which indicate the extent to which each element of the multivariate dataset contributes to the LV. In the case of PLS for the FFT amplitude spectrum, the permutation test, indicated whether one or more of the LVs was statistically significant, the Design Scores indicated whether this was a main effect of STATE, GROUP or an interaction between the two, and the saliences indicated the frequencies and electrode channels where the effects were seen.

As a secondary analysis, FFT amplitudes were compared using a mixed design ANOVA with STATE (Hypnosis vs. Pre-hypnosis) and REGION (Region Left Frontal, Left Central, Left Posterior, Right Frontal, Right Central and Right Posterior) as within-subject measures and GROUP (High susceptibles vs. Low susceptibles) as a between subject measure. For REGION, FFT amplitude values were averaged as follows (Left Frontal: FP1, F7, F3, FC5; Right Frontal FP2, F8, F4, FC6; Left Central: T7, C3 CP5, CP1; Right Central: T8, C4, CP6 CP2; Left Posterior: P7, P3, PO1, O1; Right Posterior: P8, P4, PO2, O2).

#### EEG functional connectivity

The COH and iCOH data for all 378 electrode pairs for each STATE (Hypnosis vs. Pre-hypnosis) and GROUP (High susceptibles vs. Low susceptibles) were analyzed using PLS. Separate analyses were conducted for each frequency band (Delta, Theta, Alpha, Beta1, Beta2, and Gamma). In the case of PLS for the COH and iCOH, the permutation test, indicated whether one or more of the LVs were statistically significant, the Design Scores indicated whether this was a main effect of STATE, GROUP or an interaction between the two, and the saliences indicated the electrode pairs where the effects were seen.

For all PLS analyses, the statistical significance was determined using permutation testing with 1000 permutations, and the reliability of the saliences (i.e., where and weighting of the Latent Variable was significantly greater than zero) was established using bootstrapping with 1000 re-samplings.

## Results

There were no significant differences in the EEG amplitude spectrum between the pre-hypnosis and hypnosis conditions or between the high and low susceptible groups (PLS: LV1, 76.71% of the crossblock covariance, *p* < 0.303; LV2 23.29%, *p* < 0.985). This was confirmed by the ANOVA (Table 1) which showed that there were no significant effects of STATE, GROUP or interaction between STATE, GROUP, and REGION in any frequency band. In short, there was no evidence of any change in EEG amplitude between the pre-hypnosis and hypnosis conditions.

**Table 1:** **Results of the ANOVA on the EEG amplitude by STATE, GROUP, and REGION**.

	**State**	**Group**	**State × Group**	**State × Region**	**State × Region × Group**
Delta	*F*_(1, 21)_ = 0.040, *p* < 0.843	*F*_(1, 21)_ = 2.189, *p* < 0.154	*F*_(1, 21)_ = 0.071, *p* < 0.793	*F*_(5, 17)_ = 0.727, *p* < 0.612	*F*_(5, 17)_ = 1.397, *p* < 0.275
Theta	*F*_(1, 21)_ = 0.377, *p* < 0.546	*F*_(1, 21)_ = 2.828, *p* < 0.107	*F*_(1, 21)_ = 0.255, *p* < 0.619	*F*_(5, 17)_ = 1.091, *p* < 0.401	*F*_(5, 17)_ = 0.850, *p* < 0.533
Alpha	*F*_(1, 21)_ = 0.060, *p* < 0.808	*F*_(1, 21)_ = 2.220, *p* < 0.151	*F*_(1, 21)_ = 0.029, *p* < 0.867	*F*_(5, 17)_ = 0.847, *p* < 0.535	*F*_(5, 17)_ = 1.367, *p* < 0.285
Beta1	*F*_(1, 21)_ = 0.0000, *p* < 1.000	*F*_(1, 21)_ = 1.287, *p* < 0.269	*F*_(1, 21)_ = 0.603, *p* < 0.446	*F*_(5, 17)_ = 2.230, *p* < 0.099	*F*_(5, 17)_ = 0.176, *p* < 0.968
Beta2	*F*_(1, 21)_ = 0.816, *p* < 0.377	*F*_(1, 21)_ = 1.670, *p* < 0.210	*F*_(1, 21)_ = 1.844, *p* < 0.189	*F*_(5, 17)_ = 2.341, *p* < 0.086	*F*_(5, 17)_ = 0.632, *p* < 0.678
Gamma	*F*_(1, 21)_ = 0.629, *p* < 0.437	*F*_(1, 21)_ = 0.854, *p* < 0.366	*F*_(1, 21)_ = 0.689, *p* < 0.416	*F*_(5, 17)_ = 1.420, *p* < 0.267	*F*_(5, 17)_ = 0.299, *p* < 0.907

*State: Hypnosis vs. Pre-hypnosis; Group: High susceptibles vs. Low susceptibles, Region Left Frontal, Left Central, Left Posterior, Right Frontal, Right Central and Right Posterior*.

There were also no differences in functional connectivity results between the pre-hypnosis and hypnosis conditions or between the high and low susceptible groups using COH in any frequency band (Table 2). Only in the Theta frequency band was a trend toward statistical significance for the first latent variable (PLS: LV1, 65.99% of the crossblock covariance, *p* < 0.071) and examination of the Design Scores showed that this was a LV that contrasted the pre-hypnosis and hypnosis conditions in both groups. That is, there was a non-significant trend toward theta coherence being higher in the hypnosis state than in the pre-hypnosis state for both groups of participants.

**Table 2:** **Results of the Partial Least Squares Analysis of Coherence/Imaginary Coherence by state (Hypnosis vs. Pre-hypnosis) and group (“High Susceptibles” vs. “Low Susceptibles”)**.

	**Coherence (% crossblock variance, permutation test *p*-value)**		**Imaginary Coherence (% crossblock variance, permutation test *p*-value)**	
	**1st Latent variable**	**2nd Latent variable**	**1st Latent variable**	**2nd Latent variable**
Delta	56.51%, *p* < 0.358	43.49%, *p* < 0.736	55.74%, *p* < 0.439	44.26%, *p* < 0.904
Theta	65.99%, *p* < 0.071	34.01%, *p* < 0.832	**63.17%, *p* <0.013^a^**	36.83%, *p* < 0.880
Alpha	50.98%, *p* < 0.661	49.02%, *p* < 0.687	60.48%, *p* < 0.343	39.52%, *p* < 0.962
Beta1	59.14%, *p* < 0.390	40.86%, *p* < 0.886	**61.57%, *p*< 0.043^b^**	38.43%, *p* < 0.865
Beta2	54.71%, *p* < 0.347	45.29%, *p* < 0.640	57.85%, *p* < 0.071	42.15%, *p* < 0.681
Gamma	52.86%, *p* < 0.529	47.14%, *p* < 0.728	67.99%, *p* < 0.230	32.01%, *p* < 0.931

a *See (Figure 2A) to show the Design Scores associated with this result*.

b *See (Figure 3A) to show the Design Scores associated with this result*.

There were, however, significant differences in the theta and beta1 frequency bands for iCOH (Table 2). For theta, LV1 was significant (63.15% of the crossblock covariance, *p* < 0.013) and the Design Scores (Figure 2A) showed that this effect was a contrast between the pre-hypnosis and hypnosis conditions for the high susceptibles only. Those functional connections where there was a significant difference in iCOH between the Hypnosis and Pre-hypnosis conditions are shown in (Figure 2B). The changes were predominantly an increase in iCOH in the hypnosis condition compared with the pre-hypnosis condition that clustered at central posterior sites with a maximum at Pz (Figure 2C).

**Figure 2:**
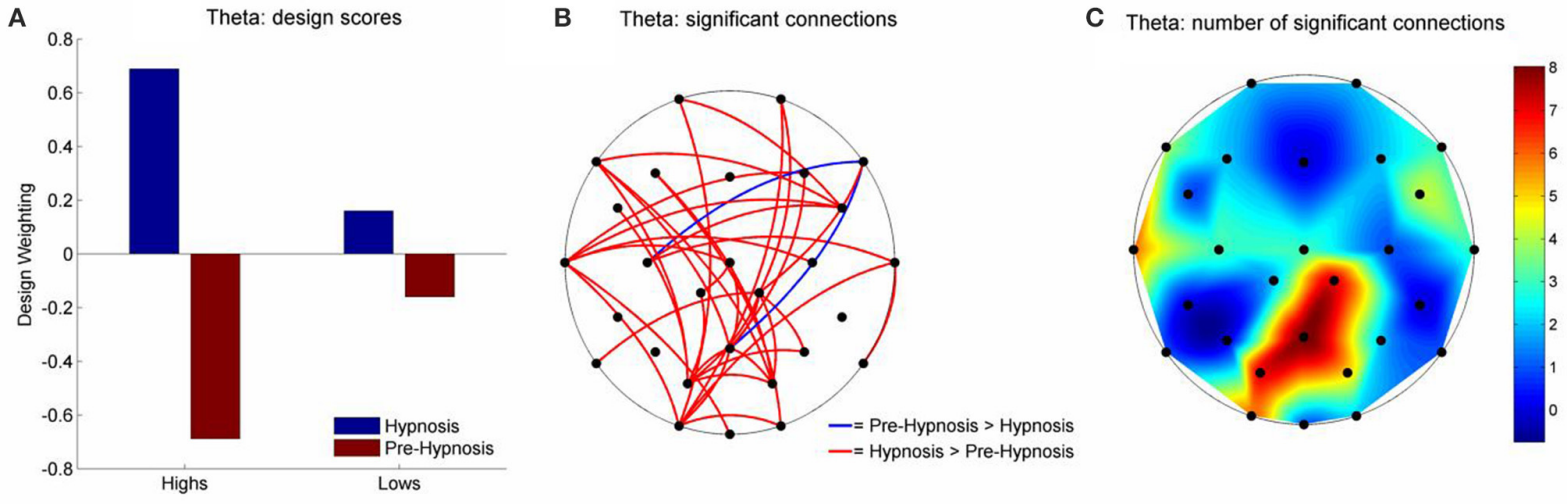
**Schematic representation of the change in iCOH between the pre-hypnosis and hypnosis states in Theta. (A)** Shows the Design Scores for the significant LV which was a contrast between the pre-hypnosis and hypnosis states predominantly for the high susceptible participants. **(B)** Shows those connections that significantly loaded on the LV, red lines showing a positive loading and blue lines showing a negative loading. Given the Design Scores, the red lines indicate those connections where there was an increase in iCOH from the pre-hypnosis state to the hypnosis state in the high susceptible participants; Blue lines indicate those connections where there was a decrease. **(C)** Shows the number of significant changes in iCOH associated with each electrode site. In this case, there was a hub of connections focused on the central-parietal region that was maximal at Pz.

**Figure 3:**
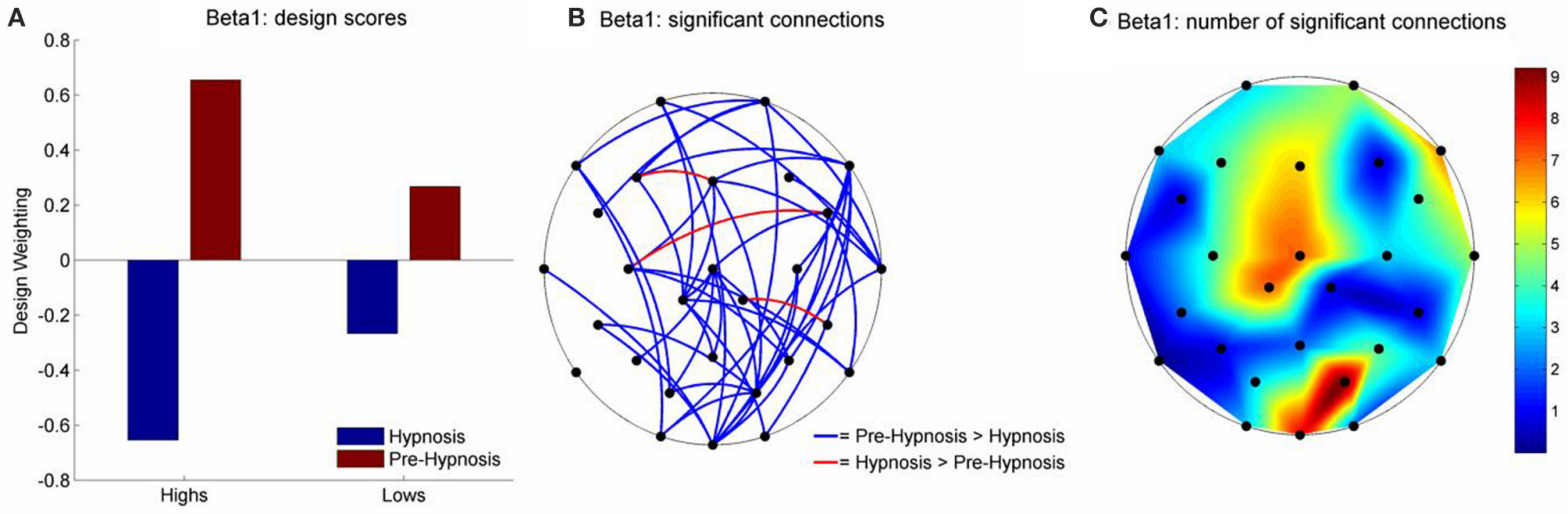
**Schematic Representation of the change in iCOH between the pre-hypnosis and hypnosis states in Beta1**. **(A)** Shows the Design Scores for the significant LV which was a contrast between the pre-hypnosis and hypnosis states. The loadings were greater for the high susceptible than for the low susceptible participants but the relative difference was less strong than was seen in Theta (Figure 2A). **(B)** Shows those connections that significantly loaded on the LV, red lines showing a positive loading and blue lines showing a negative loading. Given the Design Scores, the blue lines indicate those connections where there was a decrease in iCOH from the pre-hypnosis state to the hypnosis state and red lines indicate an increase in iCOH. The differences were most pronounced in the high susceptible participants. **(C)** Shows the number of significant connections at each electrode site. In this case, there were two clusters of connections; one centered on the vertex (maximal at Cz), and one focused at posterior electrodes (maximal at PO2).

For beta1, LV1 was significant (61.57% of the crossblock covariance, *p* < 0.043) and the Design Scores (Figure 3A) showed that this effect, like that for theta, was a contrast between the pre-hypnosis and hypnosis conditions. Again, the contrast was strongest for the high susceptibles but, the weightings of the low susceptibles were somewhat stronger than for theta. Those functional connections where there was a significant difference in iCOH between the Hypnosis and Pre-hypnosis conditions are shown in (Figure 3B). The changes were predominantly a decrease in iCOH in the hypnosis condition compared with the pre-hypnosis condition that clustered at fronto-central and occipital sites (Figure 3C).

**Figure 4:**
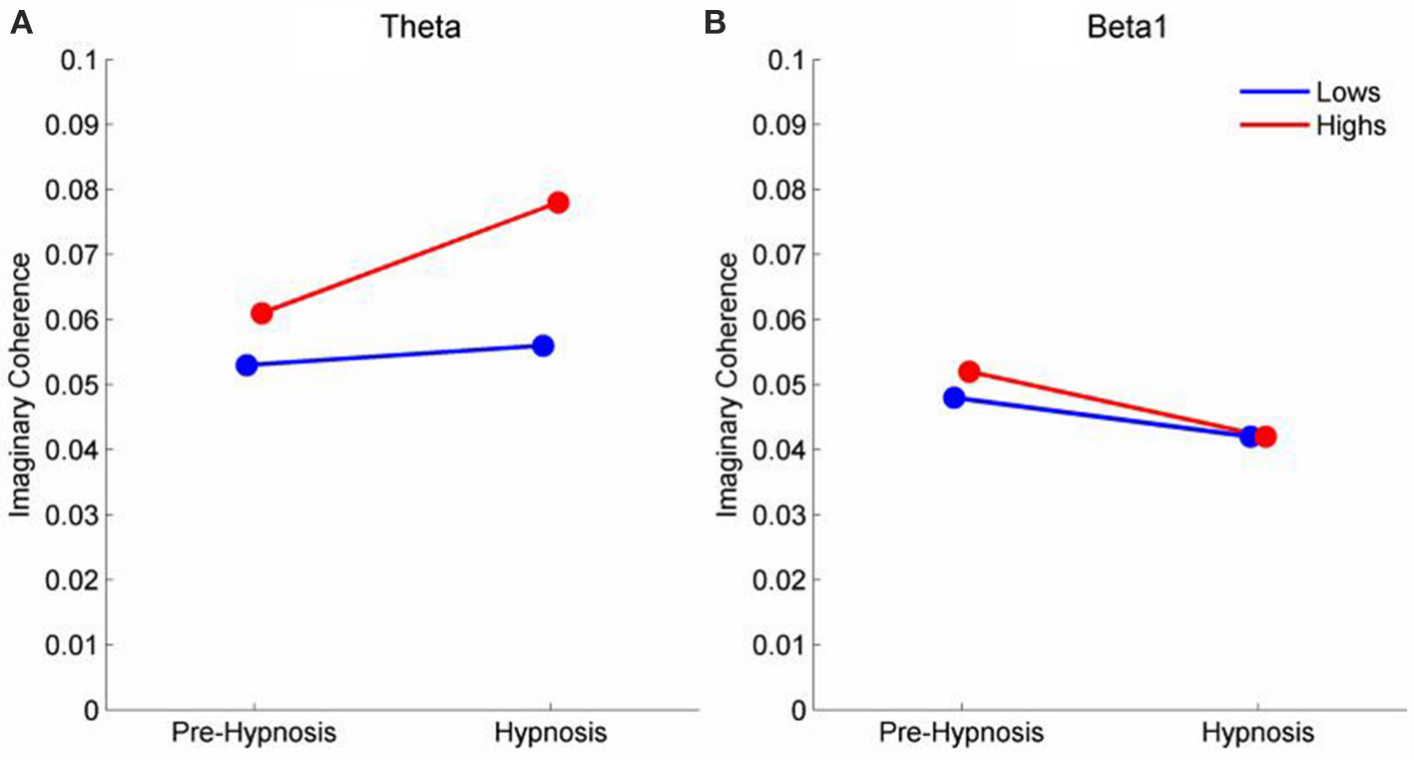
**Mean iCOH in the Hypnosis and Pre-hypnosis conditions for high and low susceptible participants in the **(A)** theta and **(B)** beta frequency ranges**.

The mean change in iCOH for those connections that differed significantly on the PLS analysis are shown in (Figure 4A) shows that iCOH for the low susceptible participants did not differ between the pre-hypnosis and hypnosis conditions. Both groups of participants showed similar levels of iCOH during the pre-hypnosis condition but the high susceptible participants showed a significant increase in iCOH during hypnosis. Figure 4B shows the iCOH for beta1. Both groups show slightly lower iCOH during the hypnosis condition but the change was marginally greater for the high susceptible participants.

## Discussion

The present study has identified two candidate neurophysiological markers, for the presence of a hypnotic state. Each marker takes the form of a changed pattern of functional connectivity from a pre-hypnosis baseline to the period immediately following the hypnotic induction. The first (and stronger finding) was for increased theta band functional connectivity following hypnotic induction in high but not low susceptible participants organized around a central-parietal hub. The second was for decreased functional connectivity in the beta1 band following hypnosis stronger in the high than the low susceptible participants around fronto-central and occipital hubs.

Several other functional connectivity studies of EEG changes during a resting hypnotized condition have been published in recent years (Fingelkurts et al., 2007; Terhune et al., 2011; Cardeña et al., 2013). Each of these studies features the use of a unique functional connectivity measure seeking, as does the present study, to avoid the well-known problems of volume conduction and multiple comparisons associated with traditional coherence analysis. Using their own structural synchrony measure (Fingelkurts et al., 2007), found an increase in the number of functional connections for the theta frequency band and decrease of functional connectivity for the beta frequency band during hypnosis, however they also report significant results across all of the traditional frequency bands. Terhune et al. (2011) reported a significant decrease in high susceptibles in hypnosis in the Phase Lag Index between frontal and parietal electrode groupings in the upper alpha band whilst Cardeña et al. (2013) found topographic variability in beta and gamma band to be related to hypnotic depth reports amongst high susceptibles.

In the present study candidate markers were identified by PLS analysis of iCOH but not ordinary COH data confirming the importance of removing the effects of volume conduction in order to make functional interpretations of EEG coherence analyses. Another important feature of the present analysis was the use of PLS to identify and extract the deep structure of the EEG data specifically related to the experimental manipulations which were the necessary focus of this investigation. Given equivalent sensory and behavioral processing demands in the pre and post hypnotic induction conditions we did not expect to find any significant differences in spectral band amplitude measures between pre and post hypnotic induction, high or low susceptibles or the interaction of these factors and we did not. Therefore, changes in band amplitude do not represent a plausible alternative explanation of the present findings (Florian et al., 1998).

The possible finding of a neurophysiological marker of the hypnotic state is of the utmost importance for the development of the state vs. non-state debate in hypnosis research and for the cognitive neuroscience of hypnosis and related conditions (Hasegawa and Jamieson, 2002; Kallio and Revonsuo, 2003; Jamieson and Woody, 2007). A successful state marker will be observable whenever a hypnotic state is present and absent when it is not present making it possible for researchers to distinguish between hypnotic and non-hypnotic responses to the same suggestion. Another very important application, with potential clinical significance, is the identification of the operation of a hypnotic state, and hypnotic processes, in conditions outside of formal hypnosis where it has been hypothesized to operate, such as post-traumatic dissociation, trance or possession states, or some psychological and medical conditions. The role of hypnosis in these conditions is highly controversial. If proven a biomarker of the hypnotic state could provide a final resolution of these important issues.

At this point such applications must await future development. The first task is to robustly replicate and quasi-replicate the present results if they are not to join the graveyard of the many specific and interesting cognitive neuroscience findings in the area of hypnosis that have been reported with excitement and then neither replicated nor built upon. Mature science is not built upon individual experiments but upon programs of research where multiple experiments build upon, criticize, and feed into each other. It is a troubling feature of contemporary cognitive neuroscience research into hypnosis that this is not currently happening. In the case of the findings reported here, this issue can be readily and easily addressed. Numerous laboratories around the world have archives of pre- and post—hypnotic induction multichannel EEG data from high, low hypnotically susceptible participants and such datasets can be readily reanalyzed using the methods employed here to establish the robustness of the present results.

In particular existing coherence analyses in the domain of hypnosis could be revisited with the present techniques. Recent EEG studies of hypnosis employing alternative functional connectivity measures (e.g., Fingelkurts et al., 2007; Terhune et al., 2011; Cardeña et al., 2013) may also test the robustness of these findings by applying the present methods to their data sets while the current dataset could be similarly reanalyzed with those alternative measures to determine if similar results are obtained. This would require active cooperation across many laboratories and the sharing of raw data sets. Such a development would greatly facilitate both the replication and the testing of network related hypotheses in this area and might usefully lead to the establishment of a repository of hypnosis neuroscience datasets (EEG, MEG, fMRI, PET, etc.) of past and present studies, updated as new datasets (of both published and unpublished studies) become available. We believe this should be a priority task for the future.

Beyond the necessity of replication it remains essential to further understand the nature of the functional neurophysiological system/s which underlies the present results. Are there two independent function networks involved, one corresponding to the theta findings and the other corresponding to the beta 1 findings? Or do they interact? Or are they rather both expressions of a deeper underlying process? Although these two candidate markers occur in different frequency bands, they might reasonably be considered to reflect complementary features of a single process. Indeed, the re-configuration of cortical oscillations across conventional frequency boundaries may be much more common (and necessary) than once thought (Canolty and Knight, 2010) and it has recently been proposed as a potential mechanism to account for both induced and evoked changes in the EEG (see the Firefly model Burgess, 2012). However, at this point we simply do not know, but we will need to know if the concept of a hypnotic state is to acquire further scientific understanding. A useful step to explore in this direction may be to take the analysis of these resting state functional connectivity changes from sensor space (in this case recording electrodes) to source space (estimated reconstruction of oscillatory activity at cortical gray matter sources) and to examine changes in connectivity between the estimated sources.

### Functional roles of theta and beta1 networks

While there are few direct parallels between the present findings and recent cognitive neuroscience studies of hypnosis there may be some points of contact that give a clue to the possible functional meaning of the current results. Looking first at the LV1 results for theta we see that the iCOH *increases* in hypnosis appear to be organized around a central-parietal hub (see Figure 2C). Functional connectivity in the theta band has been closely linked to the coordination of transient functional coupling (exchange of information) between distant cortical regions (Von Stein and Sarnthein, 2000; Schack et al., 2005). The repeatedly observed phenomena of gamma-theta nesting (Burgess and Ali, 2002) provides a mechanism allowing long range theta synchronization to coordinate bottom-up processing activity in widely separated local networks at the specific time points as required by controlled cognitive processing (Womelsdorf et al., 2010).

Synchronized theta oscillations have been shown to play a key role in active cognitive processes including episodic memory (Burgess and Gruzelier, 1997, 2000; Nyhus and Curran, 2010), working memory (Sauseng et al., 2010), error detection (Cohen, 2011) and semantic processing (Sauseng et al., 2005). Each of these cognitive operations is associated with the experience of deliberate effortful control, the very antithesis of the experience reported by high susceptibles when responding to hypnotic suggestion (Polito et al., 2013). Theta elicited in these contexts is characterized by a topography known as frontal midline theta and is closely associated with the operation of top down attentional processes of cognitive control (Mitchell et al., 2008). For this reason evidence of attentional modulation by hypnotic suggestion (Egner and Raz, 2007) and sporadic reports of enhanced theta activity in high susceptibles, in hypnosis or both (not found in the present study) is widely interpreted as evidence that the engagement of executive attentional control lies at the heart of hypnotic phenomena. By contrast, contemporary dissociation theories of hypnosis (Jamieson and Woody, 2007; Sadler and Woody, 2010) point to evidence for a breakdown in the coordination of frontal executive control in hypnosis (Jamieson and Sheehan, 2004; Egner et al., 2005) as indicating that a fundamental reorganization of higher level control processes is being implemented in the hypnotized brain.

It is apparent that the hypnosis-related increases in theta connectivity shown by the high susceptibles in our study did not show the fronto-central hub associated with frontal midline theta and executive attention control (see Figure 2). This finding may be reflected in the fMRI study of resting hypnosis by McGeown et al. (2009) who report a deactivation in the rostral division of the ACC in high susceptibles following hypnotic induction. Rather the theta connectivity increases in the present study clustered around a central-parietal hub. This aspect of the current findings may also be reflected in a previous fMRI study of responses to a hypnotic paralysis suggestion for the left hand (Cojan et al., 2009). When required to respond subjects showed increased activity in the right motor cortex (despite paralysis) indicating a preparatory motor intention to respond. Coincident with this activation increased in the precuneus (central-parietal cortex) as did functional connectivity with right motor cortex. Cojan et al. (2009) suggest that their findings may indicate the role of (hypnotically suggested) high level self-representations operating through a parietal attention mechanism in orchestrating and coordinating the behavioral response to this suggestion.

Looking next at the LV1 results for beta 1 we see that the topography of iCOH *decreases* in hypnosis appear to be organized around a fronto-central hub followed by an occipital hub (see Figure 3C). While great caution must be applied to any inference from sensor (electrode) space to cortical source space this first hub overlies motor and premotor cortex and supplementary motor areas. Intracortical recording studies from homologous regions in awake monkeys have uncovered the major role played by beta oscillations in maintaining motor activity throughout large scale motor networks (Brovelli et al., 2004). In addition (Bosman et al., 2012) have shown that cortical beta primarily originates from the same deep cortical layers from which feedback projections arise (while fast frequency gamma sources lie primarily in shallow layers from which feed-forward projections arise). Blakemore et al. (2003) have provided compelling evidence to support the theory that the perceived involuntariness of responses to hypnotic ideomotor suggestions are due to a failure of the premotor cortex to generate “efference copies” of motor commands leading to inaccurate forward models of self-generated actions which in turn has been shown to underlie the experience of involuntariness found in hypnotic ideomotor suggestions (Blakemore et al., 2003). In a recent fMRI study (Deeley et al., 2013) found that loss of perceived control of movement by high susceptibles responding to hypnotic suggestion was directly related to decreased functional connectivity between the supplementary motor area and components of the wider motor system (including the occipital/visual cortex). The possibility of a relationship between the current beta1 iCOH findings and these studies is entirely speculative but one may reasonably suggest that, if future research is conducted into similar hypnotic suggestions from an electrophysiological perspective, then the investigators should consider examining the role of beta1 band functional connectivity.

### Limitations of this study

An important limitation of the current study is that the design does not counterbalance the order hypnotic and non-hypnotic testing conditions and therefore it cannot rule out the possibility of order effects unrelated to the administration of the hypnotic induction causing the observed iCOH changes between pre and post hypnotic induction eyes closed resting EEG recordings. Cardeña et al. (2013) sought to control for this possibility by using repeated testing at intervals within the hypnosis condition while Williams and Gruzelier (2001) and Jamieson et al. (2005) utilized an ABA design conducting non-hypnotic testing in both pre and post hypnotic testing periods. The latter two studies found separate effects in the pre hypnosis vs. hypnosis conditions to those in the hypnosis vs. post hypnosis conditions and we suspect such temporal order (but genuine) hypnosis effects are an intrinsic feature of hypnosis itself. Order effects, as an alternative explanation, do not identify a specific cause of results but rather describe a feature of an unknown causal mechanism. Two features of the current results make a non-hypnosis related order effect an unlikely explanation for the pre-post hypnotic induction effects observed. The first is that these differences are systematically related to hypnotic susceptibility. As noted previously the major criterion for designation the effect of a suggestion administered in hypnosis (and the hypnotic induction may be considered as the first such suggestion) as “hypnotic” is that it is systematically related to hypnotic susceptibility. The second, though related to the first, is that these effects are larger in those with high hypnotic susceptibility than those with low susceptibility. Plausible non-hypnotic time related psychological processes such as boredom, distraction and random thought processes might plausibly be expected to be greater in low than high susceptible participants so that if anything time related differences related to these processes would be greater in the low susceptible group. However, we consider it prudent for future research to systematically manipulate testing order to confirm or eliminate the presence of treatment (hypnotic induction) unrelated order dependent effects. We note that the common practice of collapsing results across order counterbalanced conditions at best smears the effect of any treatment unrelated order effect and at worst mixes two independent asymmetric order related non treatment mechanisms and so does not provide an adequate control for such order effects (Jamieson et al., 2005).

Future evaluation of the present findings must take into account the potential role of specific suggestions included in different hypnotic induction procedures (although present data were derived from a period following the induction rather than during the induction itself). While we have taken the important step of identifying a candidate neurophysiological marker for the hypnotic state the neural foundations of such a state (which may or may not be the same thing as a neural marker for the state, although they must at the very least be related) will play a direct role in accounting for key features of the changed phenomenology which has hitherto been the primary basis for attributing the existence of such a state. This has not yet been demonstrated in the present study and must await the application of appropriate phenomenological measures and analysis in conjunction with quantification of the currently proposed hypnotic state markers in future studies (Pekala and Kumar, 2000, 2007; Pekala, 2002; Deeley et al., 2012; Cardeña et al., 2013).

As cogently noted by McGeown et al. (2009) altered state theories of hypnosis do not merely posit that an altered state is one of the outcomes of hypnosis but that the nature of the altered state plays at the very least an enabling role in the emergence of those responses to specific hypnotic suggestions that may truly be called hypnotic. It must be acknowledged that at most the present work demonstrates that hypnosis is accompanied by an altered state of neural network organization and not that this state plays a role in responding to the different types of hypnotic suggestion (ideomotor, motor-inhibition, perceptual-cognitive and amnesia) that are increasingly the focus of cognitive neuroscience studies (Oakley and Halligan, 2013). However, it is a priori most implausible that such a major functional reorganization of interactions between and within neural networks will have no implications for ongoing cognitive processes. Having a reliable marker for hypnotic state, as we have proposed here, is a crucial first step. Once it can be determined that we have found such a marker the causal dynamics of the hypnotic state can begin to be unraveled.

## Author contributions

Adrian P. Burgess and Helen J. Crawford designed the study and collected the data. All the hypnotic inductions and the assessments of hypnotic susceptibility were conducted by Helen J. Crawford. Adrian P. Burgess and Graham A. Jamieson analyzed the data and wrote the paper.

### Conflict of interest statement

The authors declare that the research was conducted in the absence of any commercial or financial relationships that could be construed as a potential conflict of interest.
